# Surgical Operations upon the Ovaries

**Published:** 1891-04

**Authors:** 


### SURGICAL OPERATIONS UPON THE OVARIES.

(Transactions of I. H. A., Morning Session, June 26th, 1890.)

Dr. J. B. Bell—The day before yesterday it became my duty to assist a young colleague, Dr. Emerson, in a laparotomy. I did not see the patient until she was on the table. The operation was the removal of the uterine appendages for the relief of annoying and dangerous reflex symptoms, and was beautifully and skillfully performed. It is sometimes called Tait's or Battey's operation, and consists in the complete removal of the ovaries and other appendages of the uterus, without the excuse of large tumors and abnormal growths, for the purpose of giving relief to numerous symptoms of ill-health, depending, or supposed to depend, upon the diseased conditions of these organs. It is an open question whether the operation is ever justifiable or not. Surgery is the opprobrium of medicine. There should be no surgery except that small amount rendered necessary by accidents. There should be no tumors, no cancers to remove, but we have not yet reached our ideal in surgery, or medicine, or obstetrics, and we cannot accomplish all we desire. Poor people cannot wait for a careful scientific study of their case and a perfect cure which would come in time. They have to take what they can get, and we have to give them such prompt, if imperfect, relief as we can.

Of course, as homœopaths we do not want and do not need Battey's operation, but do we as surgeons? The case in question was a young girl who suffered so greatly from menstruation as to be incapacitated from the ordinary duties of life. Our young friend, the doctor, believed that the removal of something from the pelvis might help her. The specimen I here show you is the ovary and its tube.

I do not believe she will be cured by the operation, and the question is, whether it is proper and advisable to remove the ovaries for dysmenorrhœa. The ovary, as you see, is cystically degenerated, and in course of time would probably have become a large tumor. The operation is more frequently required in salpingitis and pyo-salpingitis; their most frequent cause being gonorrhœal infection.

Noeggerath was the first to point out the danger of gonorrhœa being transmitted from a man to his wife and giving rise to deep-seated and serious affections, thus confirming our doctrine that the malady is not at all due to the gonococcus, but to an internal miasm or virus. These views have been generally accepted by the old school, but they do not seem to have received much help thereby as regards treatment. The question is, whether in cases of deep-seated pelvic trouble from the above or any other cause, with numerous reflex symptoms, where treatment has been carried on for some time unsuccessfully we are justified in removing the ovaries and appendages.

The removal of the uterine appendages for the cure of fibroids is also a question on which there is much difference of opinion in the old school. The object of the operation is to bring about an early menopause. It is often unsuccessful, and sometimes progresses very rapidly to a fatal issue. Even when successful, so far as producing a cessation of the catamenia goes, it often brings on a state of ill-health, with an increase of the distressing symptoms peculiar to the climacteric period. The woman is then harder to cure than before the operation.

Hence, the old school has called a halt, more or less. At least the trend of the discussion is that way. Our remedies are usually able to tide over the patient until the natural arrival of the menopause.

Besides the ovary I have here a small cyst. Surgically I approve of this ovary having been removed, but homœopathically I cannot.

Dr. Winn—The patient spoken of by Dr. Bell underwent the operation for a severe retroflexion. It was questioned at the time, whether the ovary was not prolapsed upon the uterus. I was present at the examination just before the operation. I have been reading Tait the last few days, and find that he advises in these cases two modes of treatment. Where the patients are wealthy, and surrounded by the comforts of life, he advises conservative hygienic treatment, especially complete rest during the menstrual flow, but where the patient is poor and dependent upon her daily labor for a livelihood he advises early surgical interference, as the most satisfactory.

Dr. Bell—In the operation for the relief of pelvic pain, caused by adhesions, the idea is very apparent, but as a rule the adhesions reform, and the relief is only temporary.

Dr. Winn—Tait speaks very strongly against the use of the pessary. He says it is a useless practice, and generally makes the case worse. He also directs that the uterus should not be straightened, but let alone in its mal-position.

Dr. Adams—In a case of a woman suffering with a large fibroid and excessive hemorrhages, relief was obtained under the use of Lil-tig.$^{cm}$. The monthly sickness did not stop, but became normal in character.

Dr. Hawley—It seems to me the surgeons give up the question. They say, practically, that these conditions are curable under homœopathic treatment—only give them time enough, but the poor girl cannot wait so long, she has no place to stay, therefore we will remove the ovaries.

Now it would not cost any of us much in dollars and cents to treat that poor girl for a year or two, or three, and I, for one, would do it, and I could find friends enough who would pay her lodgings. It is the doctor's first duty to cure the sick, not to cut them to pieces, besides, when the operation is performed, the opportunity of curing has probably been lost.

I have had, within a month, a young woman under my charge, married, and a mother, who had been for three years in the care of the gynecologists, with the idea which they had put in her head, that she had an ovarian tumor. I do not know whether she had a tumor or not; if so, the ovary was not larger than a walnut, but I do know that she is now free from pain, and so happy she does not know what to do with herself, under a little homœopathic treatment.

Dr. Fincke—I cured, during the last two years, a tumor in the ovarian region with Lachesis$^{cm}$, no trace of it now remaining.

Dr. Thompson—Four years ago I was called to see a case of uterine fibroid in a lady about the menopause. She had been under many physicians, both allopathic and homœopathic. I also had the diagnosis of one of the Professors of Bellevue College.

It seemed to me to be a case of progressive, pernicious anemia. There seemed to be very few blood corpuscles in her system. She looked horrible; like a corpse. After an examination by the surgeon he stated that she might live a month, or six weeks, and that an operation, on account of the poverty of the blood would be impossible. I have been treating her four years, and she is better than she has been for ten years; goes about the house and does a good deal of her work. Her lips are red and the tumor, which was growing rapidly, has ceased to grow.

When a tumor is ten, fifty, or a hundred pounds, I believe an operation may be necessary, to relieve the patient of the great mechanical weight; but not in ordinary cases.

Dr. Baylies—I had a case of fibroid tumor that was diagnosed by Dr. J. C. Miner, of New York, in conjunction with myself. I treated the case, and was successful in the course of two years. The remedy was Bellad.$^{90m}$.

Dr. Stone—I have had three cases like that of Dr. Bell, in which the consulting physicians advised ovariotomy. The first operated upon had both ovaries removed in a degenerated condition. She had a very slow convalescence, and I cannot see that she is much better than she was before.

The second also had both ovaries removed, and has now fully as much trouble as before. We had counsel, Dr. Packard, of Boston, and she is now going to consult Dr. Thomas, of New York, probably with the expectation of finding something more to remove.

The third had only one ovary removed at first, and in a year the second one, but she still menstruates, and has had severe flowing spells since. She is to-day a worse sufferer than before the first operation.

This being my experience, I have felt considerable dislike for this operation. I greatly prefer treatment with homœopathic remedies.

Dr. Hawley—Dr. Bell has expressed the idea that the woman could have been cured under proper homœopathic treatment. I would like to ask him if the operation has not acted as a bar to the proper cure of the case. Can she ever be cured homœopathically, since the surgical interference ?

Dr. H. C. Allen—No.

Dr. J. B. Bell—I do not believe that I would have operated in this case, but I do not say that I would never do it in an appropriate case, and I do not think that an appropriate case would ever come from good homœopathic hands. I do not know whether such an operation prevents the homœopathic cure or not.

Dr. H. C. Allen—The most frequent cause of this trouble is the gonorrhœal poison, as has been shown in many able articles written on the gonorrhœal infection and its effects upon the ovaries and tubes. The old school, while they have given us admirable descriptions of these troubles, are impotent to cure; they drop the matter at the description of it. If they only knew the power that lies in Thuja and Medorrhinum how much better they would get along. Irritable ovary and fibroid tumors can be relieved by the similar remedy. I agree with Dr. Bell that in our own number we do not see these cases. Six months' treatment before the operation would have done away with the necessity for the operation.

Seven or eight years ago the wife of a homœopathic physician, now in Iowa, was taken ill. Her case was diagnosed by Dr. Ormes as fibroid tumor. This diagnosis was confirmed by many physicians, both old and new school.

She was sent to an institution where she could have electrical treatment, and returned worse than she was before. Dr. Ormes said that nothing but removal of the uterus and ovaries would cure her. Her husband wrote to me to get my opinion as to who was the best ovariotomist. Dr. Porter, whom I recommended, confirmed the previous diagnosis, and said the ovary was as large as a cocoanut. She had severe hemorrhages. She decided that when she died she would take her uterus and ovaries with her.

Under the action of two remedies in six months she was pregnant, and at the seventh month of pregnancy was delivered of a two and one-half pound boy. In two years she was delivered of a healthy child weighing eight pounds, and in four years there could be found no trace of a tumor of any kind.

The remedies indicated and used successively were Psorinum $^{42m}$ and Conium $^{70m}$.

Dr. Hawley—About a month ago I treated a lady, whose physician had found, three years ago this spring, a tumor in the region of the left ovary. She went from Nebraska to New York and saw Dr. Thomas, who after an examination confirmed the idea of a tumor, and advised her to wait until the following autumn before submitting to an operation. During this interval a sister of hers, who was a patient of mine, advised her to consult me. I prescribed for her by letter and there was very soon a reduction in the growth, and two months later pregnancy announced itself. The physician attending her proposed to produce an abortion because he had the idea in his head that the woman could not be delivered with that tumor there. She went safely through the labor, however, and the tumor was discovered to be no bigger than a hen's egg. It had been much larger. I saw her lately and she was as well as she ever was in her life. I cannot remember for certain what her remedy was, but think it was Psorinum $^{4m}$ (F.).

Dr. Dever—A lady who had been injured in the right ovary was told by Dr. Franklin that nothing under the sun would relieve her but an operation. I prescribed Conium for her and she was cured by it.

Dr. Fincke—I should like to call on the surgeons to give us their idea of the physiological use of the ovaries and tubes.

Dr. Stow—I suppose the nearest we can get to this question is this: The uterus as well as the ovaries are concerned in menstruation. At the time of the escape of the ovum from its follicle the uterus is engorged with blood, and from its lining membrane exudes the blood which escapes with the ovum. Now there becomes established, from constant repetition of this, a habit of becoming congested at certain times, of so strong a nature that this turgescence continues to recur even when the ovaries have been removed. It is a peculiar attendant upon the normal function of the uterus, continuing even after the extirpation of the very organs upon which that function depends. There is a rapidly growing tendency on the part of both old and new-school surgeons to operate in cases where there is no necessity for operating. This formidable operation has actually been performed many times for the cure of headache. I have known of several such cases, but the headache is not cured, only modified.

A surgeon in Syracuse has performed ovariotomy five or six times for the relief of occipital pains. He claimed there was no other permanent cure of the trouble. In three of them the headache continued, and in one was slightly lessened. Such cases as have been cited here are simply beautiful, and we need more such to offset this growing tendency to extend the field of surgery to where it does not belong. The necessity for the existence of this Hahnemannian Association was never more apparent than now, when the old school are performing this operation for every female complaint. We need to combat them; we need to show that the operation is not necessary. We need to impress upon the women of this country that they can be cured of these conditions by Homœopathy without operation. It is our mission to spread these ideas as widely as possible.

